# Cluster-based assessment of protein-protein interaction confidence

**DOI:** 10.1186/1471-2105-13-262

**Published:** 2012-10-10

**Authors:** Atanas Kamburov, Arndt Grossmann, Ralf Herwig, Ulrich Stelzl

**Affiliations:** 1 Department of Vertebrate Genomics, Max Planck Institute for Molecular Genetics, 14195 Berlin, Ihnestr. 63-73, Germany; 2 Otto-Warburg Laboratory, Max Planck Institute for Molecular Genetics, 14195 Berlin, Ihnestr. 63-73, Germany

## Abstract

**Background:**

Protein-protein interaction networks are key to a systems-level understanding of cellular biology. However, interaction data can contain a considerable fraction of false positives. Several methods have been proposed to assess the confidence of individual interactions. Most of them require the integration of additional data like protein expression and interaction homology information. While being certainly useful, such additional data are not always available and may introduce additional bias and ambiguity.

**Results:**

We propose a novel, network topology based interaction confidence assessment method called CAPPIC (cluster-based assessment of protein-protein interaction confidence). It exploits the network’s inherent modular architecture for assessing the confidence of individual interactions. Our method determines algorithmic parameters intrinsically and does not require any parameter input or reference sets for confidence scoring.

**Conclusions:**

On the basis of five yeast and two human physical interactome maps inferred using different techniques, we show that CAPPIC reliably assesses interaction confidence and its performance compares well to other approaches that are also based on network topology. The confidence score correlates with the agreement in localization and biological process annotations of interacting proteins. Moreover, it corroborates experimental evidence of physical interactions. Our method is not limited to physical interactome maps as we exemplify with a large yeast genetic interaction network. An implementation of CAPPIC is available at http://intscore.molgen.mpg.de.

## Background

Accurate interaction networks (interactomes) are fundamental to answering questions about how the biochemical machinery of cells organizes matter, processes information, and carries out transformations to perform specific functions leading to various phenotypes. Toward this goal, a number of experimental [1] and computational [2-4] techniques have been devised and applied to map the interactions of human proteins [5-8] and those of model organisms such as yeast [9-12]. Despite their incompleteness [13], current interactome maps already serve as a basis for numerous methods aiming to elucidate biological processes in health and disease [14,15]. Current interactome maps are contaminated with false positive interactions that can make up a considerable portion of the data [13,16-20]. These false positive interactions dim the explanatory light of interaction networks and also decrease the predictive value of methods using such data. It is thus of primary importance to derive confidence values for individual interactions, which can serve to refine current interactome maps or can be used as interaction weights. For example, it has been shown recently that the performance of complex detection approaches is better in confidence-weighted protein-protein interaction networks than in non-weighted networks [19,21].

Several approaches have been proposed for interaction confidence assessment, many of which are reviewed in [19,22,23]. Most of these methods integrate additional data like interaction homology [17], co-expression of genes encoding interacting proteins [17,24,25], or a combination of these and other evidence features [26,27]. The outcome from such methods depends on the additional data sets. Others combine multiple topological features with additional knowledge to achieve better predictions [20,28]. Methods which are able to use network topology alone to predict interaction veracity [29-32] are the tools of choice for interaction confidence assessment if other types of data are limited or biased.

At various levels (globally as well as locally), the topology of interaction networks encodes biological properties which are largely independent of the biochemical function of the individual members of the network [33,34]. This has been demonstrated through analysis of global properties exploiting topological features such as node degree [35] or distance [36,37]. The biological importance of network topology may be even more clear for local structures, as in the case of specific wiring patterns of interaction partners [34]. Likewise, modularity of interaction networks is currently the most successful concept for addressing the dynamics of cellular processes [8,38,39].

Goldberg and Roth [29] proposed a connectivity based approach for interaction confidence assessment where the number of common neighbors of a pair of predicted interaction partners counts in support of the interaction. They defined interaction confidence as the level of enrichment of common network neighbors of interacting proteins. It is quantified by the hypergeometric distribution P-value given the number of common neighbors and total network neighbors of both interacting proteins. The underlying principle of the approach has been established in seminal studies demonstrating that biological networks are marked with short interaction paths separating random pairs of proteins in the network (small-world property), and densely connected local neighborhoods (neighborhood cohesiveness property) [40]. Real protein-protein interactions are expected to meet the network cohesiveness property more frequently than false positives. More recently, Kuchaiev and co-authors [32] proposed another method that embeds interaction networks into a low-dimensional Euclidean space based on network metrics (shortest path length) and then calculates confidence of interactions depending on the Euclidean distance between proteins within that space. The basis of the approach is the geometric graph model that was proposed to better reflect biological networks than e.g. the small-world model [41]. Although the biological basis of the geometric graph model remains elusive, the authors show that it measures network distance more reliably. Both of these topology based methods assign confidence as numerical values to protein-protein interactions in a network and are additionally able to predict new interaction candidates by assigning confidence scores to non-interactions. However, both methods have certain shortcomings. The method by Goldberg and Roth is able to assess the confidence of those interactions whose participants have common neighbors only. Often, however, interacting proteins do not share neighbors. The method of Kuchaiev *et al.* appears limited in that it requires fixing six free parameters. These include algorithm-specific parameters as well as the prior probability for interactions which depends on knowledge about the interactome size.

Here, we propose CAPPIC (cluster-based assessment of protein-protein interaction confidence) – a novel approach that exploits the inherent modular structure of interactomes for confidence assessment of protein-protein interactions. Our method combines the basic principles of the topology based methods described above: high neighborhood interconnectedness of a couple of proteins and short distance between them (the features exploited by Goldberg and Roth and Kuchaiev *et al.*, respectively) are indicators that both proteins participate in the same module. We apply Markov clustering [42] to the line graph [43] of an interaction network to dissect it into modules of interactions. As demonstrated in [44], this strategy can generate interaction clusters that significantly overlap with known biological pathways. Notably, the interaction clusters overlap in their protein constitution. This is biologically more meaningful than clustering the proteins into disjoint modules because pathways and protein machineries are known to overlap [10,21]. The rationale behind our approach is that proteins that are specific to certain modules are expected to have more interactions with proteins that are specific to the same modules than with other proteins [39]. Intuitively, we assign low confidence to interactions that disagree with the modular structure of biological networks and high confidence to those that comply with it. This rationale has also been used as a basis of approaches for the detection of binary interactions [10] or protein complexes [45] from complex purification data or to reveal dynamic interaction patterns during the human spliceosome cycle [8]. While the aim of CAPPIC is to detect false positive interactions, a different approach, which is however also based on the principle of high link density within network modules, has been proposed for identifying false negatives [46].

We applied our method to six large-scale interaction networks from yeast to assess its performance and compare it to previous topology-based methods (Table 1). The six networks were fundamentally different with respect to their biological and topological properties as they have been generated using different techniques. These included: 1) a network that was generated using the protein-fragment complementation assay (PCA) technology [12] (*Tarassov-all*); 2) a sub-network of Tarassov-all obtained by the authors after applying several filtering steps [12] (*Tarassov-hq*); 3) a combined network of interactions found by yeast-two-hybrid (Y2H) screens (*Yu-Ito-Uetz*) comprising the networks published by Yu *et al.*[9], Ito *et al.*[47] and Uetz *et al.*[48] (the integrated data set was retrieved from [9]); 4) a network of interactions predicted by Collins *et al.*[49] from protein complex data resulting from affinity purification assays coupled to mass spectrometry (AP-MS) [10,11] (*Collins*), downloaded from BioGRID [50]; 5) a comprehensive physical interaction network from the interaction meta-database ConsensusPathDB, release 6(yeast) [51] obtained by the integration of multiple publicly accessible interaction repositories (CPDB-yeast); and 6) a genetic interaction map published by Costanzo *et al.*[52] obtained at a stringent experimental cutoff (*Costanzo*). The physical interaction networks constitute a representative benchmark since they result from different, major interaction detection techniques: yeast-two-hybrid, protein-fragment complementation, affinity purification, and integration of interaction data obtained with different methods. We applied our method additionally to the genetic interaction map by Costanzo *et al.* to provide evidence that it is not limited to physical interactome maps. To show that CAPPIC’s performance was consistent across taxonomic species, we also applied it to two human networks. The first was obtained by merging the 15 largest, high-quality human yeast-two-hybrid data sets including refs. [5-8] (Additional file 1: Table S1) (*Y2H-human*). The second network corresponded to the top 5% interactions from a probabilistic binary data set generated by Mazloom *et al.*[53] from mass spectrometry-based analysis of 3,290 immuno-precipitation experiments [54] (*Mazloom*). The properties of the two human networks are summarized in Additional file 2: Table S2.

**Table 1:** Yeast interactome maps used in this study for method evaluation


network property	Tarassov-all	Tarassov-hq	Yu-Ito-Uetz	Collins	CPDB-yeast	Costanzo
references	[12]	[12]	[9]	[49]	[51]	[52]
method	PCA	PCA	Y2H	AP-MS	multiple	genetic
node count	2238 (2293)	889 (1124)	1647 (2018)	1002 (1620)	6073 (6075)	4278 (4278)
link count	9360 (9646)	2407 (2770)	2518 (2930)	8313 (9064)	74332 (74333)	63927 (63927)
clustering	0.14	0.24	0.08	0.72	0.19	0.06
coefficient						
links in	5861 (62%)	1761 (73%)	440 (17%)	8129 (97%)	63385 (85%)	47822 (74%)
triangles						
mean shortest	3.7	5.6	5.6	5.5	2.7	2.9
path length						
links with ≥ 3	546 (5%)	419 (17%)	598 (23%)	1635 (19%)	6324 (8%)	2546 (3%)
publications						

An implementation of CAPPIC is available as a web-based tool called IntScore at http://intscore.molgen.mpg.de[55].

## Results

### Approach

#### Assessing protein interaction confidence by random walk interaction clustering

**Figure 1:**
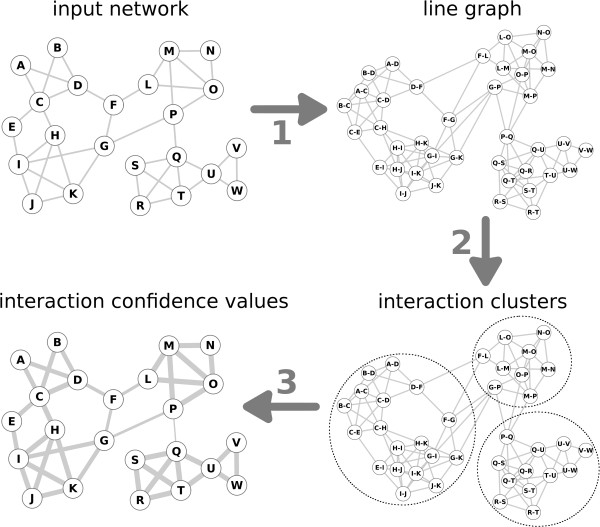
**Outline of our interaction confidence assessment method.** In the input interaction network (upper left picture), proteins are labeled with letters (A, B, etc.) and interactions between them are represented by edges. In the first step of the approach, we create the line graph of the given network where nodes represent interactions (labeled A–C, A–D, etc.) and edges represent shared interaction participants. In the second step, we use Markov clustering on this line graph to dissect it into interaction clusters. The clustering granularity is optimized in a previous step of the algorithm. Importantly, proteins can be part of more than one cluster. The relative number of interactions of a protein in a cluster determines how specific a protein is to that cluster. In the third step, we calculate confidence values for every interaction based on how specific both proteins are to the respective clusters. The thickness of interaction links in the lower left picture corresponds to the calculated interaction confidence values for this example network.

Interaction data are usually modeled as graphs where nodes represent proteins or genes and edges represent interactions between them. For assessing the confidence of every interaction in a network, we apply the following strategy (illustrated in Figure 1). First, the interaction graph is transformed into its line graph [43] where interactions are represented by nodes, and proteins are represented by links that connect their interactions (step 1 in Figure 1). Second, we deploy Markov clustering – an algorithm for network clustering through random walk simulation [42] – on the line graph to dissect it into disjoint clusters of interactions (step 2 in Figure 1). In the third and last step of the approach (step 3 in Figure 1), we evaluate the distribution of interactions among the resulting clusters. It is a key point that interactions of a given protein can be clustered together, or distributed among multiple clusters. A protein is specific to a cluster if the cluster is enriched in interactions of that protein. To quantify this enrichment, we define the fidelity *F*_*p*,*c*_ of a protein *p* to cluster *c* as the value of the cumulative hypergeometric distribution function (Equation 1) given *L*_*p*,*c*_, the number of interactions of protein *p* in cluster *c*; *L*_*p*,·_, the total number of interactions of *p* (called the degree of *p*); *L*_·,*c*_, the total number of interactions in *c*; and *L*_·,·_, the total number of interactions in the network:

(1) $$F_{p,c}=P(X\leq L_{p,c})=\overset{L_{p,c}}{\underset{k=0}{\sum }}\frac{\left(\begin{array}{c} L_{p,\cdot }\\ \;\;k \end{array}\;\right)\left(\begin{array}{c} L_{\cdot ,\cdot }-L_{p,\cdot }\\ \;L_{\cdot ,c}-k \end{array}\right)}{\left(\begin{array}{c} L_{\cdot ,\cdot }\\ L_{\cdot ,c} \end{array}\right)}$$

The value of the fidelity *F*_*p*,*c *_lies between 0 and 1, with values near or equal to 1 if a protein *p* is specific to cluster *c*, i.e. if it has relatively many links in that cluster. For a fixed *L*_*p*,*c*_ it holds that the smaller the cluster (smaller *L*_·,*c*_), the greater the fidelity value. Finally, if all the links of two proteins lie within a cluster, the fidelity is greater for the protein with the higher degree.

We define interaction confidence as the product of the fidelity values of both interacting proteins to the cluster *c* which the interaction has been assigned to:

(2) $$\text{confidence}(l_{p_{1},p_{2}})=F_{p_{1},c}\cdot F_{p_{2},c}$$

Interactions get high confidence values if both proteins are specific to the cluster containing the interaction, and low confidence values when one or both of the proteins are not specific to the cluster.

#### Optimal clustering granularity is reliably determined through partial network rewiring

The interaction confidence scores calculated by CAPPIC are dependent on the granularity of the interaction clustering. It has been previously shown that modules in many complex networks, including protein interaction maps, are organized in a hierarchical manner [56]. Accordingly, interaction clustering can yield protein complexes, cellular machineries, pathways, or higher-order biological processes depending on the clustering granularity. To estimate the clustering granularity for a network that will result in the best discrimination between true and false interactions, we first randomly rewire a small part of the links in that network to generate a false interaction set. In the rewiring procedure, pairs of interactions are selected at random and two of the proteins are swapped so that no real interaction is reconstituted and the network stays connected. This way, two false interactions are generated for two real ones while the degree of each protein is preserved. Then, we calculate interaction confidence values of the resulting partially rewired network as described above using different inflation values. The inflation parameter of the Markov clustering algorithm essentially controls clustering granularity [42]. For every inflation value, we quantify the significance of the difference between confidence score distributions of the rewired and the remaining non-rewired links. This is done with the Wilcoxon rank-sum test under the alternative hypothesis that the confidence scores of the non-rewired links are greater than the confidence scores of the rewired links. The inflation value minimizing the Wilcoxon test P-value is considered optimal.

Experiments have shown that randomly rewiring 3% of the links in the granularity estimation procedure described above is a good choice because this yields a false interaction set of reasonable size while keeping most of the network intact. If the set of false interactions obtained through random rewiring is too small, the granularity estimation will lack statistical power, while if too many interactions are rewired, the network’s original modular structure will be altered which will affect the granularity estimate. For all networks CAPPIC was applied on, random rewiring of 1%, 3%, 5%, or 10% of the interactions yielded very similar optimal granularity estimates.

Our granularity estimation strategy builds upon the assumption that the optimal granularity value inferred from a partially rewired network instance (where both false positive and false negative rates are increased compared to the real network) is transferable to the real network. We aimed to scrutinize this reasoning and verified for all reference networks that 1) the estimated optimal granularity was rather independent of the random choice of links for rewiring; and 2) that interaction clusters were similar for the intact and the partially rewired networks clustered with the same inflation value (see Additional file 3: Supplementary Text).

### True positive interactions are assigned higher confidence than false positives

**Figure 2:**
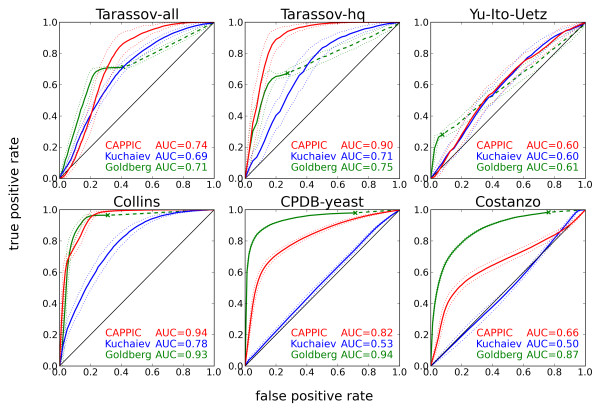
**ROC analysis measuring the performance of CAPPIC in comparison to the methods by Goldberg and Roth and Kuchaiev *et al*.** False positive rate (1-specificity) is plotted against true positive rate (sensitivity) for each of the six reference networks. Since the definition of a negative interaction set in the performance assessment involves a random process, the ROC plots summarize the outcome of 100 runs. Plots show the average ROC curves (thick lines), their standard error bands (dotted lines), as well as the mean area under the ROC curve (AUC) of all runs. The ‘X’-marks on the green ROC curves correspond to the fraction of true/false interactions whose proteins share network neighbors and are thus scored by Goldberg and Roth’s method.

We measured the performance of CAPPIC and compared it to previously proposed network topology based interaction confidence assessment methods using five yeast physical interaction networks and one genetic interactome map, covering major interaction inference methods (Table 1). We first constructed positive (literature interactions) and negative (random links) link sets and then evaluated the methods using receiver operating characteristic (ROC) analysis. The positive set for each network consisted of interactions that are reported multiple times in the literature (ranging from 3% to 23% for the six reference networks, Table 1), since such interactions have been shown to be on average more reliable [13,16]. The negative interaction set consisted of links that resulted from a random rewiring of a small sub-set (3%) of the interactions in the respective network. Interactions from the partially rewired instance, ranked with decreasing confidence value were compared successively against the positive and negative benchmark sets to determine the true positive and false positive rates at each step. In general, CAPPIC assigned higher confidence to true interactions than false interactions (Figure 2). The area under the ROC curve (AUC), which quantifies the confidence ranking performance, was as high as 94% for the Collins network. For this data set, at a fixed specificity of 80% our method reached 95% sensitivity. On the other extreme, none of the methods in the analysis showed convincing performance on the combined Y2H network Yu-Ito-Uetz. In this example, Goldberg and Roth’s method successfully classified interactions whose proteins shared network neighbors; however, such interactions comprised only 17% of Yu-Ito-Uetz (see ‘X’-mark on the green line in Figure 2 and row “links in triangles” in Table 1) while the rest of the interacting protein pairs did not share network neighbors. Goldberg and Roth’s method outperformed CAPPIC on the CPDB-yeast and Costanzo networks, whereas the method by Kuchaiev *et al.* did not discriminate (for unclear reasons) between true and false interactions better than random in these two cases. Generally, it performed worse than CAPPIC and Goldberg and Roth’s method on all networks. Based on the results for all six networks, we conclude that the method of Goldberg and Roth is able to correctly identify a subset of high-confidence interactions, but will not provide predictions for interactions not involved in triangles. On the other hand, the method by Kuchaiev *et al.* and our approach generate confidence scores for the complete data set, which is often desired when the aim is to assess the confidence of all interactions (e.g. for weighting a non-weighted network) or to filter out a relatively small sub-set of low-confidence interactions. It should be noted that in order to define a reliable negative link set, we destroyed some real interactions (increasing the false negative rate) and simultaneously introduced the same number of false positive interactions into the network. Thus, the AUC values reported here probably slightly underestimate the real performance.

In the case of well-studied organisms such as yeast, data on protein complexes can be used to define the positive interaction sets alternatively to literature evidence as used above. We used two complex-based positive sets from yeast complexes obtained from CYC2008 [57] and from ref. [58]. The performance of CAPPIC (and often of the reference methods) was better with the complex-based compared to the literature-based positive set for almost all networks (Additional file 4: Figure S1). For example, the AUC for CAPPIC increased from 82% to 87-89% for CPDB-yeast and from 66% to 70-72% for Costanzo when the literature-based positive reference set was replaced by a complex-based one; improvements by 1-2% AUC were also observed for the Tarassov-all, Tarassov-hq and Collins networks (Additional file 4: Figure S1 versus Figure 2 in the Main text). However, despite the better performance with complex-based positive reference sets, such sets are not well-suited for measuring the performance on networks obtained by techniques such as yeast-two-hybrid [9]. This could be the reason for the slight decrease in performance (by 1-2% AUC) on the Yu-Ito-Uetz yeast-two-hybrid network compared to a literature-based positive set (Additional file 4: Figure S1 versus Figure 2 in the Main text). Moreover, the complex-based performance estimate may be positively biased since protein complexes in the reference data may have been defined at least partially on the basis of the analysed interaction networks.

### Cluster based confidence scores corroborate experimental interaction evidence

**Figure 3:**
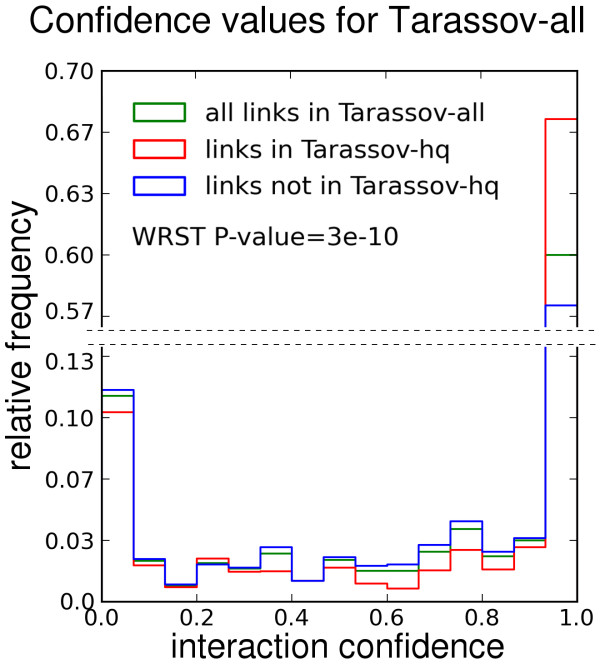
**Histogram of confidence scores for interactions in Tarassov-all calculated by our method.** The normalized histograms of interaction confidence scores are shown for the complete Tarassov-all network, as well as for its high-quality (Tarassov-hq) and non-high-quality parts. WRST: Wilcoxon rank sum test of the difference between confidence score distributions of both network parts. Note that the Y-axis is interrupted to better show the differences between the three data sets.

To compare confidence values calculated by CAPPIC with experiment-based interaction scores, we exploited the fact that some of the interactions in Tarassov-all have been designated high-quality by the authors based on experimental interaction intensity [12]. We tested whether our method assigned significantly higher confidence scores to high-quality interactions than to the rest of the interactions in Tarassov-all. As shown in Figure 3, the confidence score distributions of both interaction sub-sets were different. Using the Wilcoxon rank-sum test we confirmed that confidence values were greater for high-quality interactions than for the rest of the links in Tarassov-all (P-value <3∗10^−10^). The high agreement between cluster based interaction confidence scores and experimental interaction weight for the Tarassov-all network was corroborated by a significant Spearman rank correlation between both (*ρ *= 0.3, *p*-value < 10^−5^).

### High-confidence interactions are more consistent in biological process and cellular compartment annotation

**Figure 4:**
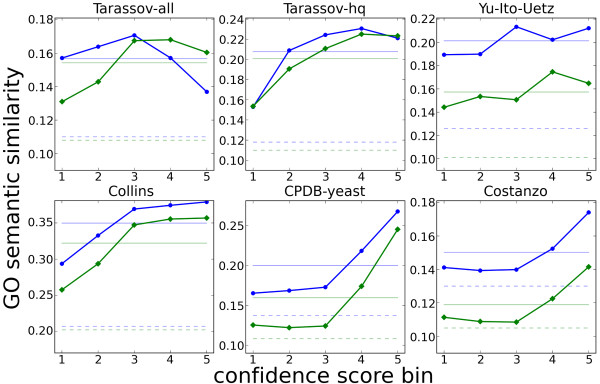
**Correlation of CAPPIC interaction confidence with semantic similarity of Gene Ontology co-annotations.** Interactions from every network are ranked by confidence and divided into five equal sized bins (X-axis); for each bin, the average semantic similarity of GO biological process (blue) and cellular component (green) annotations of interacting proteins is shown (Y-axis). Additionally, the pale continuous lines correspond to the mean GO semantic similarity over the complete network rather than the separate bins. The dashed lines reflect the average GO semantic similarity of random pairs of proteins from the network.

Interacting proteins are expected to participate in related biological processes and to be co-localized in compartments of the cell [59]. Therefore, Gene Ontology (GO) [60] annotations of interacting proteins agree more often than expected by chance. We utilized the semantic similarity of GO biological process and cellular compartment annotations of proteins predicted to interact as a performance measure of our approach. If confidence values reflect the veracity of discovered interactions, we expect interactions with higher confidence score to have a higher average semantic similarity of the proteins’ GO annotations. To test this, we ranked interactions from each reference network by confidence score and arranged them into five equal sized bins. The average GO semantic similarity (GOSemSim) values for interacting proteins in each bin are plotted in Figure 4. The GOSemSim generally correlated with interaction confidence. In several extreme cases (e.g. Costanzo), the average GOSemSim of low-confidence interactions was barely distinguishable from the average GOSemSim of random protein pairs (dashed horizontal lines), while the higher-confidence interactions reached average GOSemSim far above the average value of all interactions in the respective network (continuous horizontal lines). These results suggest that there are more false links among the lower-confidence interactions than among the higher-confidence ones.

Furthermore, if low-confidence interactions are removed from interaction clusters, the latter become more consistent regarding the pathway annotations of the contained proteins (see Additional file 3: Supplementary Text). Our approach can thus be used to obtain more refined functional modules in interaction data sets.

### The performance of CAPPIC is consistent between yeast and human networks

**Figure 5:**
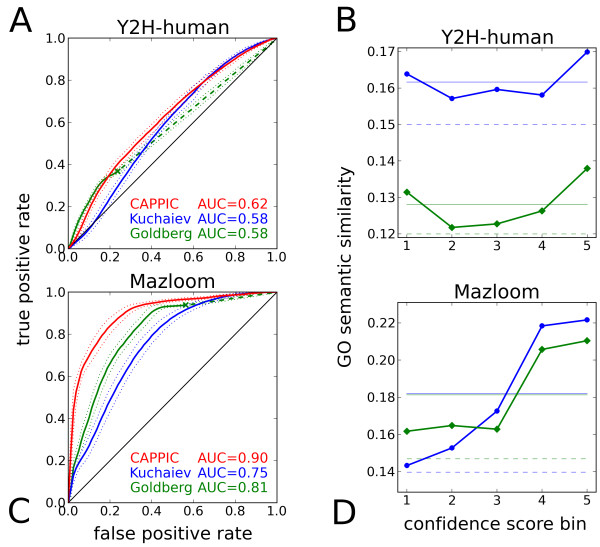
**Performance of CAPPIC on human networks.A)** and **C)**: ROC plots for Y2H-human and Mazloom, correspondingly (for details, see Figure 2 legend);**B)** and **D)**: correlation of CAPPIC scores with GO semantic similarity for Y2H-human and Mazloom, correspondingly (for details, see Figure 4 legend).

To exemplify that the performance of CAPPIC is consistent for different taxonomic species, we also applied it to two human networks: Y2H-human (Additional file 1: Table S1) and Mazloom [53]. Figure 5 shows the corresponding ROC plots summarizing the performance of CAPPIC and of the reference methods (analogous to Figure 2), as well as the GO semantic similarity as a function of the CAPPIC score (analogous to Figure 4) for these networks. Notably, the performance of CAPPIC on the Y2H-human and Mazloom human networks was very similar to the performance on the yeast counterparts obtained by analogous techniques (Yu-Ito-Uetz and Collins yeast networks, respectively). For example, CAPPIC achieved 90% AUC on the Mazloom network and 62% AUC on the much sparser yeast-two-hybrid network, outperforming the reference methods in both cases (Figure 5). In the case of the Mazloom network, we also measured the agreement between CAPPIC scores and interaction ranks that were based on evidence from 3,290 co-immunoprecipitation experiments [53]. The CAPPIC scores were calculated independently of the ranks or the confidence values assigned in the original study. The Spearman correlation coefficient between interaction ranks and CAPPIC scores was *ρ *= − 0.34 (*p*-value < 10^−5^). The correlation is negative since interactions with smaller ranks tend to get higher CAPPIC scores. As in the case of the yeast Tarassov-all network described above (that has been obtained by protein-fragment complementation assay), CAPPIC corroborates independent interaction evidence also for this human immuno-precipitation based network.

## Discussion

Network topology-based approaches are motivated by the fact that the structure of interaction networks is not random but reflects biological functionality [33,34]. Modularity is a topological property that is inherent to protein-protein interaction networks [10,39,56]. We propose a novel method (CAPPIC) to assess the confidence of individual protein interactions in an interaction network. Our method exploits network modularity alone for estimating the confidence of interactions and does not require any additional knowledge about the interacting proteins or the techniques used to generate the data. We demonstrate the power of CAPPIC in discriminating between true and false interactions on the basis of five physical protein interaction networks and one genetic interaction map from yeast, as well as two distinct interaction data sets from human.

CAPPIC compares well to previous topology-based approaches by Goldberg and Roth and Kuchaiev *et al.* in assigning continuous confidence scores to all interactions in a given physical interaction network. The method of Goldberg and Roth is dependent on shared network neighbors of interacting proteins; however, many interacting proteins do not share neighbors. As a result, many interactions are scored with a confidence value of zero. However, integrative approaches operating on networks usually take probabilistic rather than binary data as input. Thus, the goal of confidence assessment is often to assign a continuous score to all interactions rather than to filter for a small subset. In particular, all proteins with a single interaction partner are disregarded by Goldberg and Roth’s method, albeit these single protein associations could give important clues about the function of these proteins. Both methods, Kuchaiev *et al.* and CAPPIC, are able to assign continuous scores also to such interactions. In contrast to the method of Kuchaiev *et al.*, CAPPIC does not require any parameter input. The only parameter that influences the resulting confidence scores – clustering granularity – is optimized internally for each individual input network. Our results have shown that the number of clusters obtained at the optimal granularity tends to be small for all reference networks, ranging from 10 to 50 clusters (see Additional file 5: Figure S2 and Figure ST1 in Additional file 3: Supplementary Text). This alleviated our initial concerns that interactions executing essential crosstalks between related pathways could be assigned low confidence. Because the optimal granularity tends to be very coarse, closely related pathways will probably not be separated but clustered together.

CAPPIC should be applicable for weighting any binary network with an inherent modular structure (for examples, see [61]). Notably, it does not consider the technique used to generate the network (unlike other approaches that integrate a fixed, subjective judgment on the reliability of different techniques, e.g. ref. [62]). CAPPIC fails to generate reliable confidence scores in cases where modularity is not pronounced, i.e. if many of the real links within biological modules (complexes, pathways, etc.) are missing. This is probably the case with the Yu-Ito-Uetz and Y2H-human reference networks: here, the topological signal that our method exploits seems to be weaker and it achieves only 60-62% AUC. Absence of modularity in this example is evidenced by the relatively low clustering coefficient [40] of 0.08 which is nine times lower than that of the Collins network where CAPPIC achieves 94% AUC and six times lower than that of the Mazloom network (90% AUC). Moreover, the Yu-Ito-Uetz data set is the sparsest of all yeast reference networks (Table 1). To conclude, results on all example networks suggest that CAPPIC is well suited to score datasets with moderate to high interaction density.

Unlike the reference methods, CAPPIC is able to accommodate experimental evidence weights of interactions. Interaction detection techniques often associate such weights with predicted interactions, reflecting for example the number of times an interaction is observed in repetitions of a yeast-two-hybrid experiment [7,9,13] or the reporter intensity value in the case of a protein-fragment complementation assay [12]. If available, such weights can be exploited by our method in its random walk based interaction clustering step. This can improve the interaction clustering result and consequently increase the performance of confidence assessment. However, since we set out to estimate the performance of CAPPIC in comparison to other methods that cannot accommodate interaction weights, we did not make use of this advantage in this work and considered all interactions equal. Moreover, the ability to incorporate experimental interaction weights helps to avoid interaction data pre-filtering, commonly executed to derive binary interaction networks (where pairs of proteins either interact or not). Such filtering of probabilistic interaction data is inherently associated with data loss. Similarly, it is a common practice to remove interaction hubs in a dataset to improve its quality (*e.g.*, ref. [6]). As exemplified in Additional file 6: Figure S3 for the yeast hubs PHO85 (a Cyclin-dependent kinase; 467 interactions) and UBC7 (an E2 ubiquitin ligase; 622 interactions) in the CPDB-yeast network, CAPPIC assigns on average lower scores to interactions of hubs. However, a considerable fraction of their interactions scores highly: 29% of the interactions of PHO85 and 25% of the interactions of UBC7 are assigned higher CAPPIC scores than the median score of the complete network. This suggests that a complete removal of hubs from the network could unnecessarily remove high-quality protein-protein interactions and emphasizes the utility of confidence scoring.

Our approach can be combined with other lines of interaction evidence like other topological features, protein co-expression, or interaction homology to achieve even better scoring performance [22]. While the aggregation of different features holds the promise of even more reliable interaction confidence assessment, it depends on reference interaction sets. At present, even for yeast the construction of an appropriate reference set is still a daunting task [9].

## Conclusions

Since biological interaction networks contain false positives, assessing the confidence of individual interactions in order to weight or filter interaction data is a crucial step that should precede network-based inferences. Here we propose a network topology based method called CAPPIC that estimates interaction confidence by exploiting the network’s inherent modularity. CAPPIC requires no reference interaction sets or parameter settings. Based on five large-scale physical interaction networks from yeast, we show that our method compares well to other topology-based approaches. Confidence scores calculated with CAPPIC also correlate well with the Gene Ontology co-annotation of interacting proteins, and corroborate experimental evidence of physical interactions. CAPPIC is limited neither to physical interactome maps nor to yeast networks as it also performs well on a large yeast genetic interaction network and on two human protein-protein interaction data sets.

## Methods

### Application of Markov clustering algorithm

To cluster a network of interactions, we use the original implementation of the Markov clustering algorithm (version 10-201 downloaded from http://www.micans.org/mcl/sec_software.html). The inflation scan which aims to optimize clustering granularity is carried out in two steps: a coarse scan with step size of 0.1 within a fixed range *I*∈[1.1,2.0] (where *I* is the Markov clustering inflation value) is followed by a fine scan with step size of 0.025 around the optimal inflation value resulting from the coarse scan ± 0.1. In general, the inflation parameter takes values from the interval *I*∈(1.0,30.0] with higher values resulting in finer granularity. In all our experiments, the optimal inflation estimate was far below 2.0 (see Figure ST1 in Additional file 3: Supplementary Text), motivating the choice of this value as an upper boundary of the inflation scan.

### Receiver operating characteristic analysis

To conduct ROC analysis, we constructed true and false interaction sets. The positive set comprised interactions published in at least three papers in total. An exception was made for the Costanzo network because of the scarcity of genetic interaction data: the positive set in this case consisted of interactions that are also reported in [63]. Literature evidences were retrieved with the interaction evidence mining ConsensusPathDB plugin [64]. The negative interaction set was constructed by randomly rewiring 3% of the interactions in the respective network. For each partially rewired network, we ranked interactions according to confidence as calculated with CAPPIC and reference methods and created receiver operating characteristic (ROC) curves. The performance of a given confidence assessment method in ranking positive interactions higher than negative ones was quantified with the area under the ROC curve (AUC). The AUC is around 50% if a method does not perform better than random interaction ranking, and is closer to 100% the better it ranks positive interactions higher than negative ones. Since the constitution of the negative and positive sets involves a random process (that is, the random selection of interactions for rewiring), we repeated the procedure 100 times and averaged ROC results.

### Application of reference methods

We set the number of yeast genes to 6,000 in the method by Goldberg and Roth. The parameters of the method by Kuchaiev *et al.* (implemented as Matlab scripts downloaded from http://www.kuchaev.com/Denoising) were set as follows: priorEdge=0.002945 (which results when the estimated yeast interactome size of 53,000 interactions [65] is divided by the number of all possible protein pairs, 6,000 choose 2); priorNonEdge=1-priorEdge; dim=5 (default); d=3 (default); learnSetSize=min(5,000 or half the number of interactions); delta=1.0; and stopEps=0.01 (default). In the case of Costanzo, dim=3 because the program (run on a standard AMD X2 5600+ machine with 8GB of RAM running Matlab version 7.10.0.499 under Linux) did not return results within five days for a higher number of dimensions.

### Assessing semantic similarity of Gene Ontology annotations

For each network, we obtained the GO semantic similarity of biological process and cellular component annotations of interacting proteins using the method proposed by Resnik [66] implemented in the software package GOSemSim version 1.8.0 [67]. GO annotations inferred from physical interaction (GO evidence code ‘IPI’) were excluded from the semantic similarity calculation to avoid circularity. For each network, interactions were ranked by increasing confidence score and divided into five equal sized bins. The mean semantic similarity values for interacting proteins within each bin were calculated. Additionally, the mean GO semantic similarity for random pairs of proteins from the respective network was assessed by completely rewiring the networks while preserving each protein’s degree and then calculating the mean GO semantic similarity of links in those randomized networks.

## Competing interests

The authors declare that they have no competing interests.

## Authors’ contributions

AK and US conceived the method. AG and RH provided feedback on the method and contributed ideas. AK developed the method and carried out the experiments. AK and US wrote the manuscript, AG and RH provided feedback on the manuscript. All authors read and approved the manuscript.

## Supplementary Material

Additional file 1 **Table S1. **Interaction data sets merged to construct the Y2H-human network.The table lists the studies that contribute yeast-two-hybrid interactions for the merged Y2H-human network. The file is in XLS format and is viewable e.g. with LibreOffice or Microsoft Excel. Click here for file

Additional file 2 **Table S2.** Properties of the Y2H-human and Mazloom networks. The table shows the properties of the human networks used in the analysis (analogous to Table 1 in the main text). The file is in XLS format and is viewable e.g. with LibreOffice or Microsoft Excel. Click here for file

Additional file 3 **Supplementary Text.** This file contains additional text and figures demonstrating the validity of the partial random rewiring approach for clustering parameter optimization, as well as text and figures showing that CAPPIC scores can be used for interaction cluster de-noising. The file is in PDF format and is viewable e.g. with Adobe Reader. Click here for file

Additional file 4 **Figure S1.** ROC plots with complex-based positive reference sets. Receiver operating characteristic analysis results for the yeast reference networks where complex-based positive reference sets have been used. Complexes were obtained from ref. [57] (A) and from ref. [58] (B). The figure is otherwise analogous to Figure 2. Click here for file

Additional file 5 **Figure S2.** Cluster number and sizes for the yeast reference networks clustered with the optimal granularity. Yeast reference networks were clustered at the optimal inflation value into 10-50 interaction clusters. Here, the cluster sizes in terms of number of interactions (blue line, left-hand-side Y-axis) and number of genes/proteins (green line, right-hand-side Y-axis) per cluster are plotted. Click here for file

Additional file 6 **Figure S3.** Distribution of CAPPIC scores for the hubs PHO85 and UBC7 in comparison to the whole data set. Click here for file
