# Supplementary material for: Cluster-based assessment of protein-protein interaction confidence
Source: BMC Bioinformatics. 2012 Oct 10;13:262. doi: 10.1186/1471-2105-13-262 (PMC3532186; doi:10.1186/1471-2105-13-262)
Supplement: Additional file 3 — Supplementary Text. This file contains additional text and figures demonstrating the validity of the partial random rewiring approach for clustering parameter optimization, as well as text and figures showing that CAPPIC scores can be used for interaction cluster de-noising. The file is in PDF format and is viewable e.g. with Adobe Reader. [file 1471-2105-13-262-S3.pdf]

Cluster-based assessment of protein-protein interaction confidence

Atanas Kamburov, Arndt Grossmann, Ralf Herwig, Ulrich Stelzl

Supplementary text

1. Estimating optimal clustering granularity

The interaction confidence scores calculated by CAPPIC are dependent on the granularity of the interaction clustering. In order to estimate the optimal granularity for a given input network that will result in the best discrimination between true and false interactions, we create an instance of that network where a small subset (3%) of the interactions is randomly rewired, preserving each protein's degree. The estimated optimal granularity for the given network is the one that maximizes the significance of the difference between the resulting random and the remaining real interactions in the partially rewired network. This estimation builds on the assumption that the optimal granularity inferred from the partially rewired network is transferable to the original network although in the former, both the false positive and false negative rates are increased compared to the real network. To scrutinize this reasoning we tested if 1) the estimated optimal granularity was rather independent of the random choice of links for rewiring; and 2) interaction clusters were similar for the original and the partially rewired networks clustered with the same inflation.

To test the first hypothesis, we created 100 instances of each of the six reference networks (see main text) where 3% of the links were randomly selected and rewired, and performed an inflation value search for each. For every instance and every inflation value, we calculated the Wilcoxon rank-sum test P-value reflecting the significance of the score difference between original and rewired interactions (optimality criterion). The negative logarithm of the Wilcoxon test P-value and the number of clusters are plotted against varying inflation value in Figure ST1. For all six networks, the 100 randomization runs were highly consistent regarding the estimated optimal inflation value. Figure ST1 also shows that the number of clusters generated for the network instances did not vary much for any given inflation within the inflation search range.

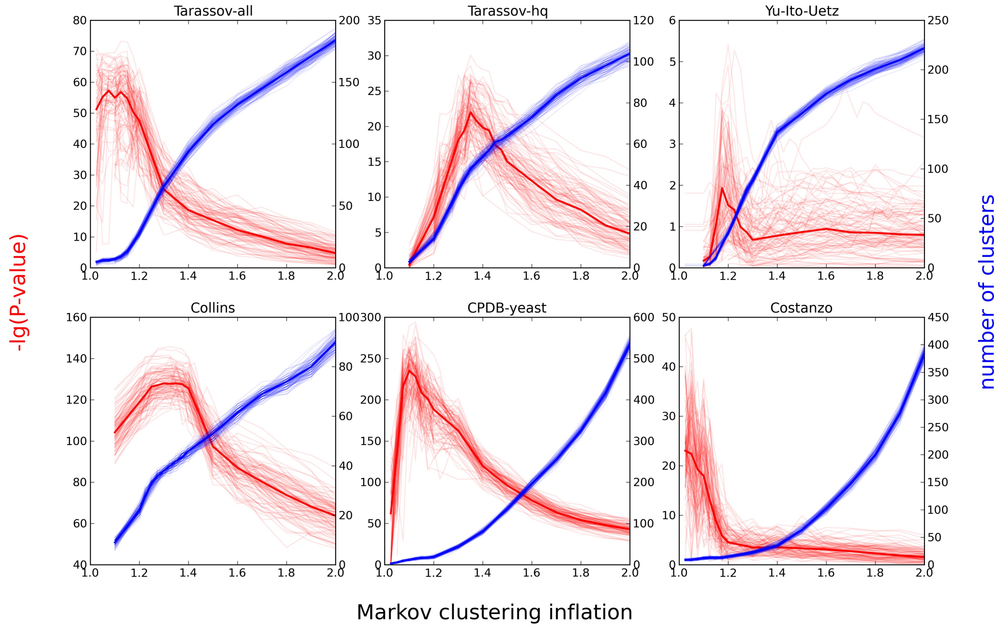

Figure ST1: Estimating optimal granularity for clustering through partial random rewiring of input networks. 100 instances of every reference network were created where 3% of the links were randomly rewired. The negative common logarithm of the Wilcoxon rank sum test P-value reflecting the confidence score difference between rewired and non-rewired interactions (red curves, left-hand-side Y-axis) was calculated for each inflation value (X-axis). Moreover, the number of resulting clusters (blue curves, right-hand-side Y-axis) is plotted against varying inflation. Thick lines indicate the median values. We note that in the case of the Yu-Ito-Uetz network, the achieved P-value in the optimization step was one to two orders of magnitude higher than for the rest of the networks. Intuitively, the reliability of confidence scores calculated by our method can be appraised from the best achieved Wilcoxon rank sum test P-value in the inflation optimization step. If the overall performance of confidence scoring for a network is bad, then the score difference between random and real interactions in the optimization phase is less significant. However, these P-values are not suited for a strict comparison between networks.

To test the second hypothesis, namely that clusters have similar interaction composition for the original and the partially rewired networks when clustered with the same inflation value, we compared the co-clustering frequencies of interactions in the non-rewired versus the rewired cases. For each reference network, we created an interaction co-clustering matrix $r_{i,j}$ which contained the relative frequencies that two interactions, i and j, end up in the same cluster for all partially rewired network instances where both interactions survive rewiring. This matrix was compared to the binary co-clustering matrix $c_{i,j}$ reflecting interaction co-clustering for the intact interaction network. We defined a clustering agreement score to measure the agreement between $r_{i,j}$ and $c_{i,j}$:

$$\text{clustering agreement} = 1 - 2\frac{\sum_{\substack{i,j \\ i\neq j}} |r_{i,j} - c_{i,j}|}{\binom{L_{\cdot,\cdot}}{2}}$$

By definition, the clustering agreement equals 1, if and only if pairs of interactions that are co-clustered in the non-rewired case are also co-clustered in all rewired instances where both interactions have survived rewiring. The agreement value is around 0 if clusters in the non-rewired and rewired instances are completely independent from each other, and equals -1 if they are negatively correlated. Figure ST2 shows the two co-clustering frequency matrices and their global mutual agreement. In all six cases we found the cluster composition of the real network in high agreement with the partially randomized networks. We conclude that clusters are very similar for the original and the partially rewired networks clustered with the same inflation value. In other words, the link randomization we introduce to estimate the optimal granularity in the clustering step of the algorithm does not change the clustering result as such.

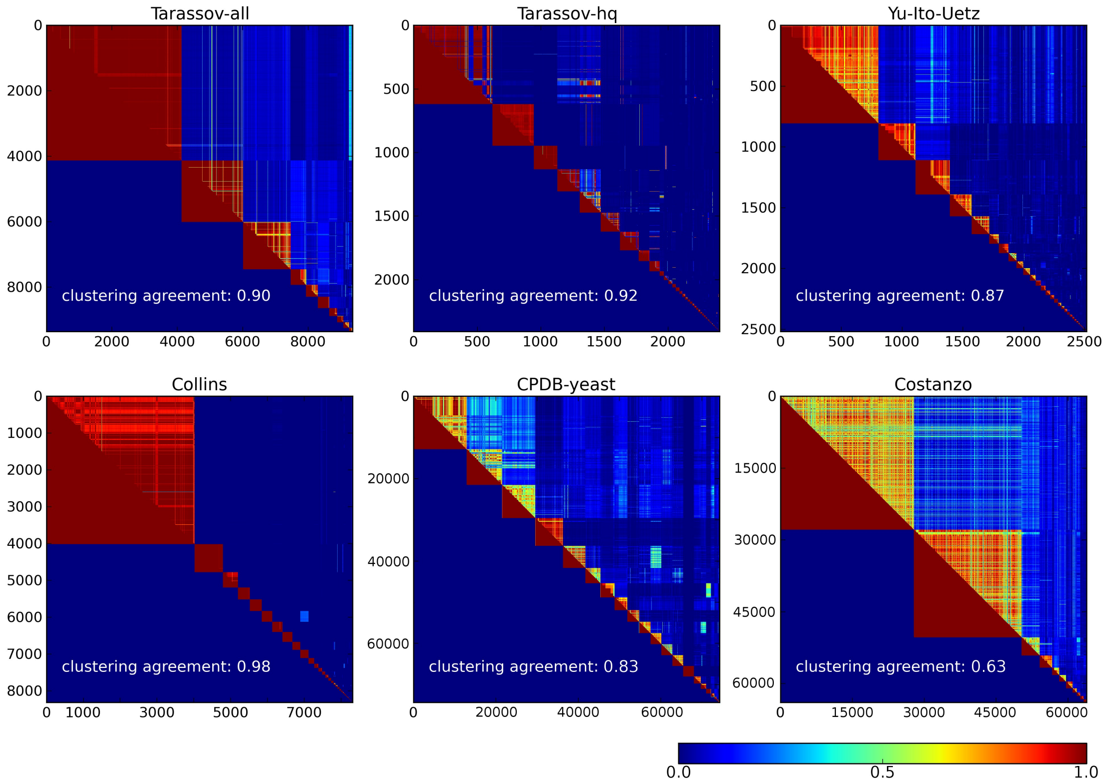

Figure ST2: Interaction co-clustering matrices. For each reference network and its 100 partially rewired instances, we calculated interaction co-clustering matrices $r_{i,j}$ and $c_{i,j}$ for a fixed inflation corresponding to the estimated optimal value. This figure shows the co-clustering heatmaps for the non-rewired networks ($c_{i,j}$, below the diagonal) and the rewired instances ($r_{i,j}$, above the diagonal); the overall agreement between both co-clustering matrices is also provided. The agreement ranges from 0.63 to 0.98 for the reference networks.

2. De-noising of interaction clusters

Pereira-Leal et al. [1] showed that Markov clustering applied to the line graph of a comprehensive interactome map yields clusters that are significantly consistent with KEGG biological pathways. Following this line of thought, we asked whether removing low-confidence interactions from the resulting interaction clusters would improve the consistency in pathway annotation of proteins remaining in the clusters. We used the scoring scheme proposed in [1] to measure the consistency of interaction clusters with KEGG pathway annotations [2] while successively removing interactions starting with the lowest-confidence ones. The cluster consistency is defined in [1] as:

$$\text{consistency} = \sum_{j=1}^{C} \left(1 - \frac{-\sum_{s=1}^{n} p_{j,s} log_2 p_{j,s}}{log_2 n} \right)$$

where C is the number of clusters computed from the line graph, $p_{j,s}$ is the relative frequency of pathway s in cluster j, and n is the number of KEGG pathways. The pathway annotation consistency of interaction clusters increased with the number of low-confidence interactions removed (Supplementary Figure ST3, black curves). Results were clearly different when the order of the removed interactions is reversed, i.e. when high-confidence interactions were removed first (dotted lines in Supplementary Figure ST3). This confirmed that lower-confidence protein-protein interactions do not fit in the pathway context of the according clusters as well as higher-confidence ones. Unlike the five physical interaction networks, in the case of the Costanzo genetic interaction map the consistency increased faster when interactions were removed from clusters starting with the high-confidence interactions. The reason for this is probably rooted in the fact that most of the detected genetic interactions involve proteins in different pathways (between-pathway interactions) than proteins in the same pathway (within-pathway interactions) [3].

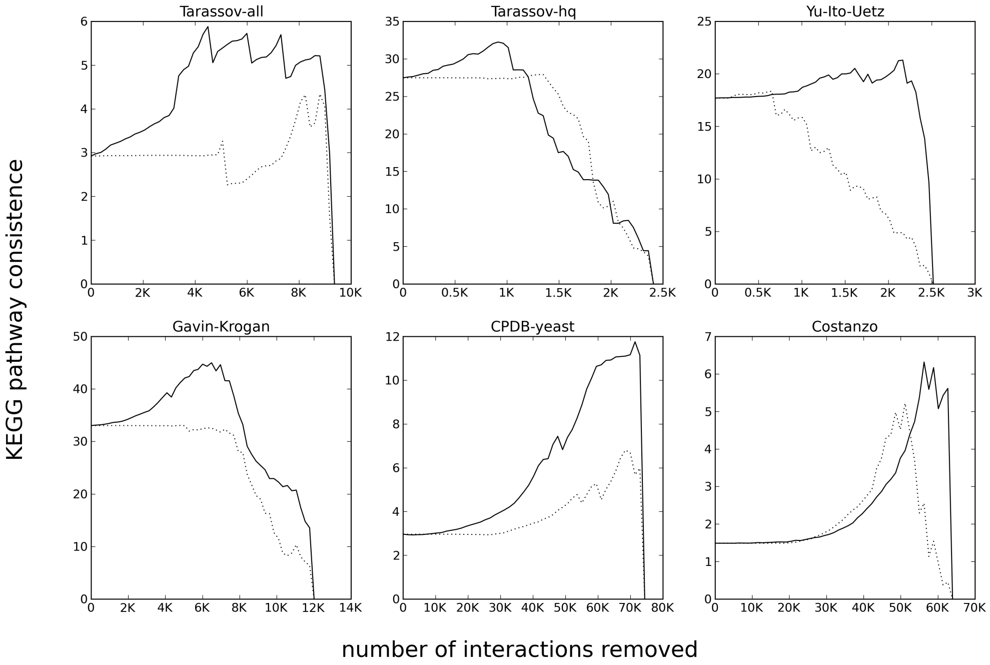

Figure ST3: Interaction cluster refinement. Each reference network was transformed into its line graph and clustered with the estimated optimal inflation value for that network. Interactions were ranked according to confidence and successively removed from the respective clusters. Pathway annotation consistency (Y-axis) was plotted against the number of interactions removed from interaction clusters (X-axis) starting with the low-confidence (continuous line) or high-confidence (dotted line) interactions.

Supplementary references

1. Pereira-Leal JB, Enright AJ, Ouzounis CA (2004) Detection of functional modules from protein interaction networks. *Proteins* **54**: 49–57.
2. Kanehisa M, Goto S, Kawashima S, Okuno Y, Hattori M (2004) The KEGG resource for deciphering the genome. *Nucleic Acids Res* **32**: D277–280.
3. Hannum G, *et al*. (2009) Genome-wide association data reveal a global map of genetic interactions among protein complexes. *PLoS Genet.* **5**:e1000782.
