# Supplementary figures and images for: Cluster-based assessment of protein-protein interaction confidence
Source: BMC Bioinformatics. 2012 Oct 10;13:262. doi: 10.1186/1471-2105-13-262 (PMC3532186; doi:10.1186/1471-2105-13-262)

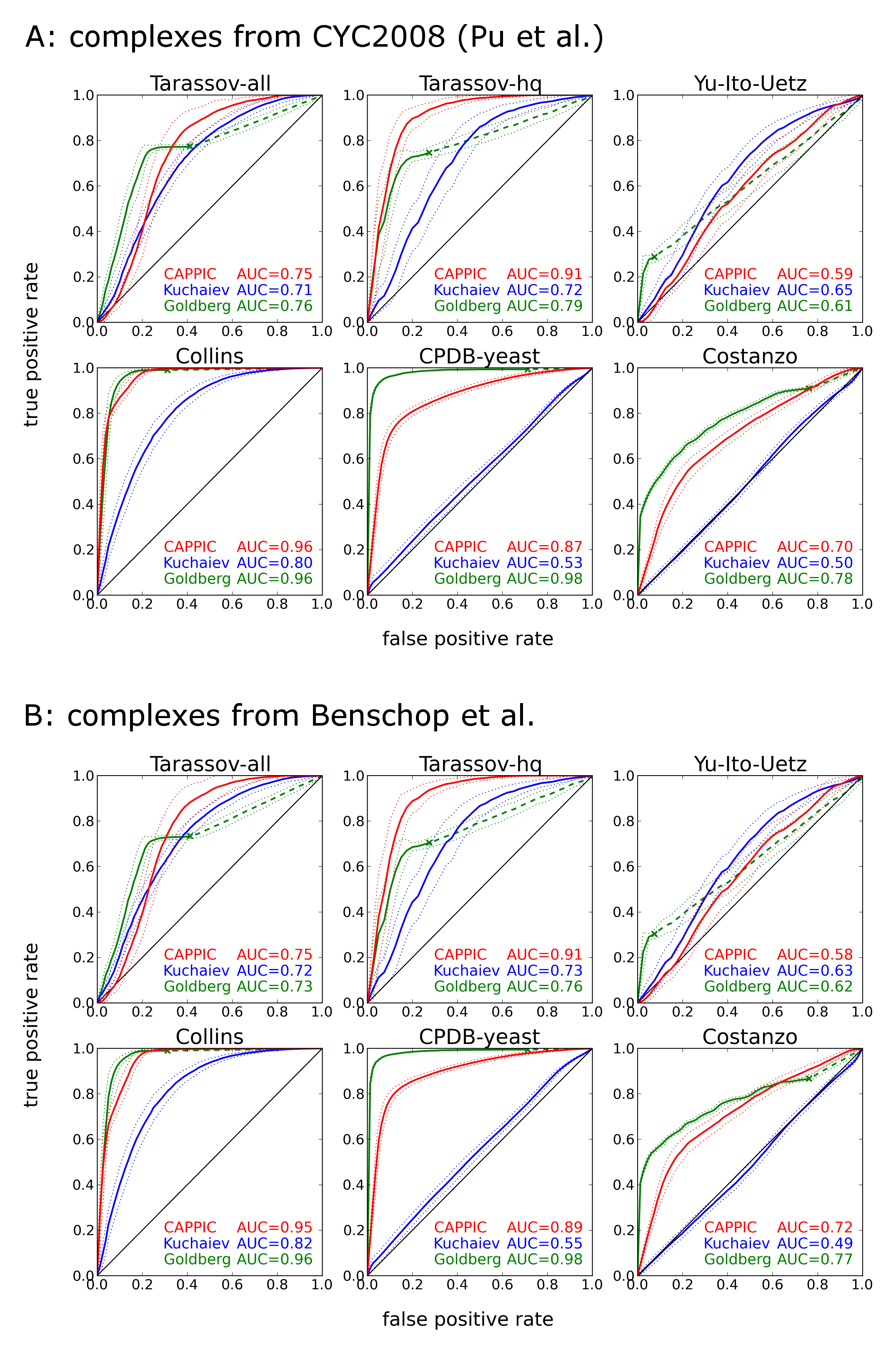

Supplement: Additional file 4 — Figure S1. ROC plots with complex-based positive reference sets. Receiver operating characteristic analysis results for the yeast reference networks where complex-based positive reference sets have been used. Complexes were obtained from ref. [57] (A) and from ref. [58] (B). The figure is otherwise analogous to Figure 2. [file 1471-2105-13-262-S4.png]

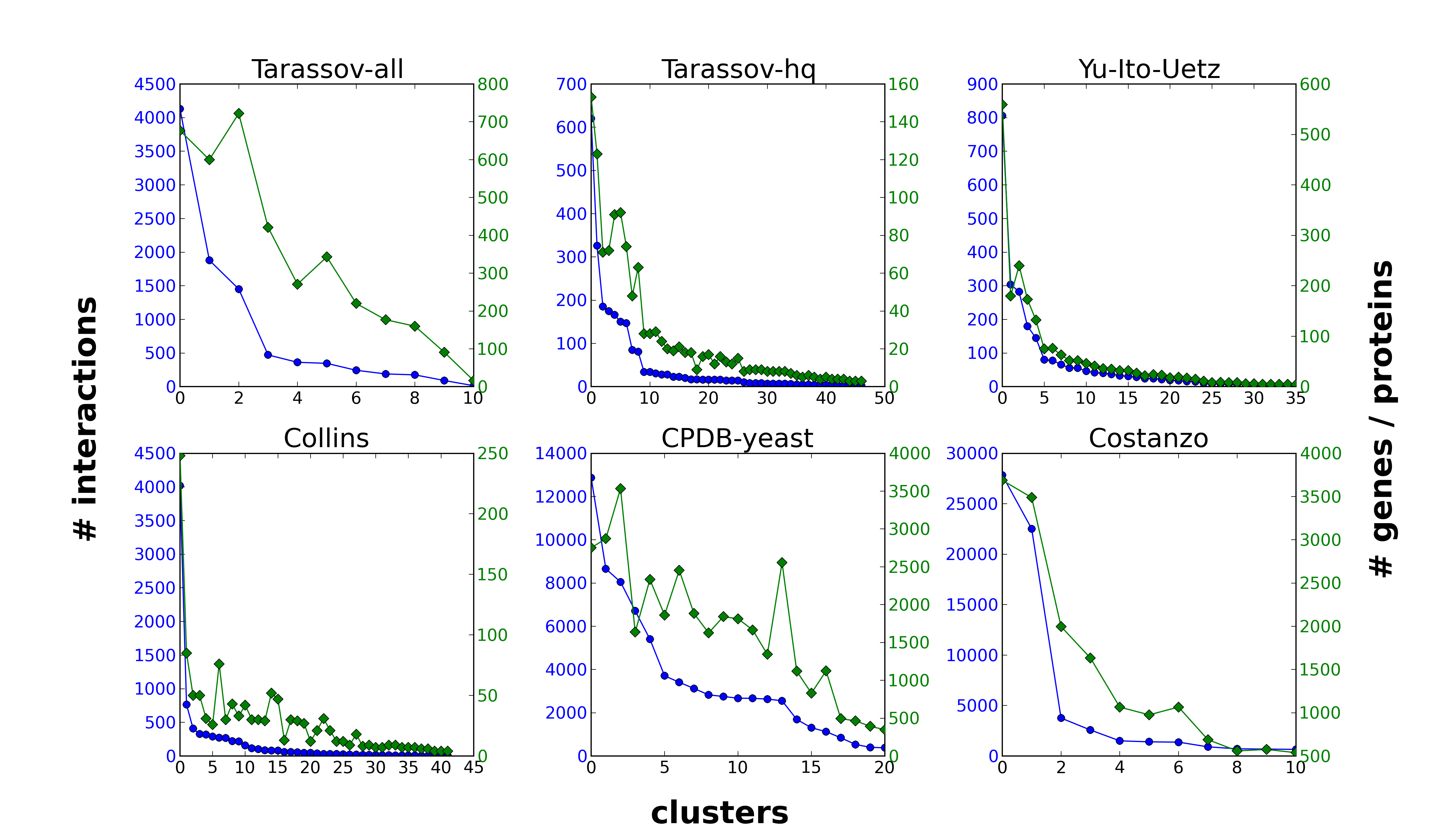

Supplement: Additional file 5 — Figure S2. Cluster number and sizes for the yeast reference networks clustered with the optimal granularity. Yeast reference networks were clustered at the optimal inflation value into 10-50 interaction clusters. Here, the cluster sizes in terms of number of interactions (blue line, left-hand-side Y-axis) and number of genes/proteins (green line, right-hand-side Y-axis) per cluster are plotted. [file 1471-2105-13-262-S5.png]

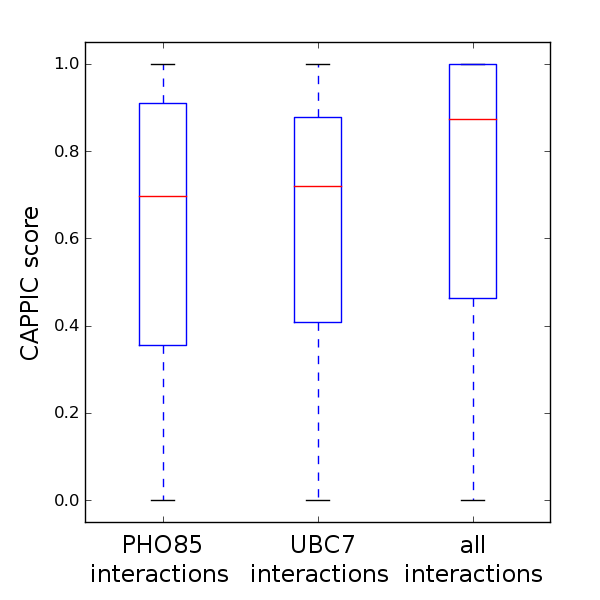

Supplement: Additional file 6 — Figure S3. Distribution of CAPPIC scores for the hubs PHO85 and UBC7 in comparison to the whole data set. [file 1471-2105-13-262-S6.png]
